# Linguistic Validation of the US National Cancer Institute’s Patient-Reported Outcomes Version of the Common Terminology Criteria for Adverse Events in Korean

**DOI:** 10.1200/JGO.18.00193

**Published:** 2019-03-27

**Authors:** Juhee Cho, Junghee Yoon, Youngha Kim, Dongryul Oh, Seok Jin Kim, Jinseok Ahn, Gee Young Suh, Seok Jin Nam, Sandra A. Mitchell

**Affiliations:** ^1^ Samsung Advanced Institute for Health Sciences and Technology, Sungkyunkwan University, Seoul, Korea; ^2^ Samsung Medical Center, Sungkyunkwan University, School of Medicine, Seoul, Korea; ^3^ National Cancer Institute, Rockville, MD

## Abstract

**PURPOSE:**

The aim of this study was to translate and linguistically validate a Korean-language version of the US National Cancer Institute’s Patient-Reported Outcomes version of the Common Terminology Criteria for Adverse Events (PRO-CTCAE).

**METHODS:**

All 124 PRO-CTCAE items were translated into Korean (PRO-CTCAE-Korean) using International Society for Pharmacoeconomics and Outcomes Research best practices and linguistically validated in a diverse sample of patients undergoing cancer treatment (n = 120) to determine whether the Korean translation captured the original concepts. During the cognitive interviews, participants first completed approximately 60 PRO-CTCAE-Korean questions and were then interviewed to evaluate the conceptual equivalence of the translation to the original PRO-CTCAE English-language source. Interview probes addressed comprehension, clarity, and ease of judgement. Three rounds of interviews were conducted. Items that met the a priori threshold of 10% or more of respondents with comprehension difficulties were considered for rephrasing and retesting.

**RESULTS:**

A majority of PRO-CTCAE-Korean items were well comprehended in round 1; 14 items posed comprehension difficulties for at least 10% of respondents in round 1. Four symptom terms (mouth and throat sores, feeling like nothing could cheer you up, frequent urination, and pain, swelling, redness at drug injection or intravenous insertion site) were revised and retested in rounds 2 and 3. For the other 10 symptom terms, no suitable alternative phrasing was identified, and the terms were retested in rounds 2 and 3. After rounds 2 and 3, no item presented difficulties in 20% or more of participants.

**CONCLUSION:**

PRO-CTCAE-Korean has been linguistically validated for use in Korean-speaking populations. Quantitative evaluation of this new measure to establish its measurement properties and responsiveness in Korean speakers undergoing cancer treatment is in progress.

## INTRODUCTION

Traditionally, adverse events (AEs) occurring in cancer clinical trials have been reported by clinicians using the Common Terminology Criteria for Adverse Events (CTCAE). Now in version 5, the CTCAE is maintained by the US National Cancer Institute (NCI).^1^ Although clinicians grade and document AEs using these standard methods, additional evaluation from the patient perspective can be valuable because approximately 10% of the AEs listed in the CTCAE are symptoms that can be best characterized by gathering information directly from patients.^2-4^ Several systematic reviews suggest that clinicians may underestimate patients’ symptom experiences, including incidence, severity, and associated distress.^5,6^ Because patient-reported outcomes (PROs) capture the patient’s perspective directly,^7,8^ they are increasingly used in both research and clinical practice.^9^ Thus, to complement clinician-based AE reporting using the CTCAE, the US NCI developed a library of PRO items (PRO-CTCAE) as a companion to the CTCAE to capture symptomatic AEs by direct patient self-report.^10,11^ Initial development of the PRO-CTCAE item library is detailed elsewhere.^12^ Of the more than 790 AEs included in the CTCAE, 78 AEs were identified as amenable to patient self-reporting. Each PRO-CTCAE item includes a plain language term for the symptomatic AE.^13^ One to three PRO items were created for each AE to evaluate the attributes of symptom presence, amount, frequency, severity, and/or interference with usual or daily activities. The standard recall period for PRO-CTCAE is “the past 7 days.”^14^ The PRO-CTCAE item library is composed of a total of 124 questions or items. In any given trial, investigators select a subset of these items for evaluation on the basis of study hypotheses, prior research, and knowledge of the anticipated regimen-related toxicities.

Because PRO-CTCAE is becoming a standardized approach for capturing symptomatic AEs in oncology trials, there exists a need to develop a language version of PRO-CTCAE that can be used with Korean speakers. The aim of this study is to translate and linguistically validate a Korean language version of the NCI’s PRO-CTCAE (PRO-CTCAE-Korean).

## METHODS

### Translation Procedure

**FIG 1:**
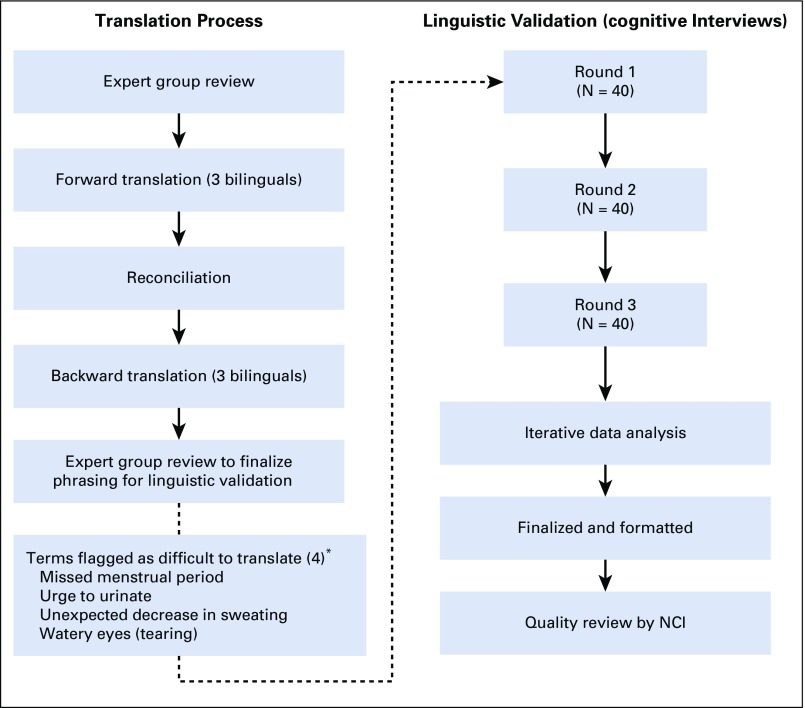
Procedure for translation and linguistic validation. (*) Symptom terms flagged as difficult to translate during the translation process were tested in the full sample of 120 participants except sex-specific questions. NCI, National Cancer Institute.

Agreement to translate the PRO-CTCAE item library into Korean was obtained from the US NCI. Figure 1 provides an overview of the translation and linguistic validation process. Three bilingual translators independently translated the 124 items, including response options, in the PRO-CTCAE item library. Translations were compared to identify semantic and conceptual inconsistencies. The comparison of the three independent forward translations revealed semantic and conceptual equivalence for 44 of 80 PRO-CTCAE symptom terms and conceptual equivalence for an additional 23 terms. For the remaining 13 symptom terms, there was minor conceptual nonequivalence. Reconciliation of terminology resulted in one final translation of each term; this term was used for the back translation carried out by three different bilingual translators. There were no identified translation issues with the response choices or the attributes of frequency, severity, interference, amount, and presence or absence.

The back translation resulted in 27 symptom terms with semantic and conceptual equivalence, 41 symptom terms with conceptual equivalence, and 10 terms with minor conceptual nonequivalence to the English-language version. Those 10 symptom terms were reviewed by the study team and Korean-speaking linguistic experts, and adjusted phrasing that was thought by the expert team to be conceptually equivalent to the English phrasing was proposed. A final review comparing the English PRO-CTCAE and the finalized back-translated Korean language version was conducted by bilingual members of the study team to identify any discrepancies and to confirm that the Korean-language phrasing was simple, grammatically correct, and likely to be comprehensible to those with lower levels of literacy or educational attainment.

Each step of the translation, back translation, reconciliation, and cognitive debriefing had oversight by a multidisciplinary expert group of native Korean speakers who were also fluent in English. The expert group consisted of two medical oncologists, two oncology nurses, two radiation oncologists, one behavioral scientist, and two health educators. The expert group reviewed the PRO-CTCAE-Korean items to identify possible linguistic and conceptual difficulties and participated in refining the phrasing to ensure item clarity, cultural relevance, and conceptual equivalence to the English item library. All documentation pertaining to the PRO-CTCAE-Korean translation, including an item history and decisions about item rephrasing, were reviewed the US NCI before the PRO-CTCAE-Korean was finalized and advanced for cognitive testing.

### Cognitive Interviewing Procedure

The PRO-CTCAE-Korean items were subsequently examined through cognitive debriefing,^15^ an interview method designed to evaluate the comprehension, ease of response, and acceptability of the terminology, phrasing, response options, and format of a newly developed PRO measure.

### Questionnaire Scripts

Using the methodology developed by the US NCI, eight Korean-language PRO-CTCAE debriefing scripts were created to guide the cognitive interviews. Each script contained a subset of approximately 60 PRO-CTCAE items reflecting approximately 30 symptomatic toxicities, as well as semistructured interview questions designed to probe the clarity, ease of judgement, and acceptability of the PRO-CTCAE-Korean phrasing (Appendix Table A1). This approach was used because the PRO-CTCAE consists of 124 individual items, and administration and probing on all items in the PRO-CTCAE item library would have been burdensome to patients undergoing cancer treatment. Prior PRO-CTCAE cognitive debriefing studies in other target languages have successfully used a similar approach.^16-18^

A subset of 15 commonly occurring cancer treatment–related symptoms was specified a priori for inclusion in all eight scripts, and thus, items reflecting these symptom terms were tested in all 120 study participants. The remaining 63 symptom terms, including five female-specific and two male-specific symptomatic AEs, were distributed across the eight scripts. In addition, four symptom terms (missed menstrual period, sudden urge to urinate, unexpected decrease in sweating, and watery eyes) had been flagged as difficult to translate into Korean by the expert group and thus were included in all scripts (as sex appropriate) and tested in all interview rounds (Fig 1). Data concerning age, marital status, educational attainment, employment status, and monthly family income, as well as physical function and overall health status, were also gathered by patient self-report. Physical function and overall health status were assessed using a subset of questions from the Medical Outcomes Study Short Form-36. Demographic and clinical information such as cancer type and stage was obtained from electronic medical records. The average time required to complete a PRO-CTCAE item was computed as a ratio of the total time (in minutes) to complete the PRO-CTCAE survey to the number of PRO-CTCAE items the participant completed. Participants who had the survey read to them rather than completing the paper-and-pencil version were excluded from this analysis. To summarize comprehension difficulties across items, a comprehension index was calculated. The comprehension index was calculated as a ratio of the number of terms (symptoms) without difficulties to the total number of symptom terms evaluated by a respondent, with higher values indicating better comprehension.

### Participants

One hundred twenty adults age 18 years or older who had been diagnosed with cancer, who were receiving either chemotherapy or radiation therapy, and who could speak, read, and write Korean as their primary language were recruited to participate in this study. Enrollment goals were prespecified to include at least 50% of participants with high school or less, 33% of participants older than age 65 years, approximately equal representation by sex, and diversity with respect to cancer site. Accrual of the sample was monitored prospectively to achieve these enrollment goals. This study was approved by the Institutional Review Board of Samsung Medical Center (Seoul, Korea), and informed consent was obtained from all study participants.

### Interviews

Three rounds of cognitive interviews were conducted; 40 participants were included in each round. Cognitive interviews were conducted in a private area of either the outpatient clinic or the Cancer Education Center and consisted of the following two parts: administration of a PRO-CTCAE survey composed of a subset of PRO-CTCAE items; and a semiscripted debriefing interview with cognitive probing of comprehension, clarity, and ease of judgement. Interviews were conducted by a graduate-level oncology nurse with experience conducting cognitive interviews in a cancer treatment setting. The interviewer was assisted by another researcher who prepared field notes.

Participants were first invited to complete a subset of PRO-CTCAE items by paper and pencil. Respondents also had the option to have the PRO-CTCAE items read to them verbatim if their physical condition limited their ability to complete the survey independently. Participants were asked to indicate those PRO-CTCAE questions that they found difficult to comprehend and those for which they had difficulty selecting a response. The interviewer did not provide any assistance or advice and encouraged patients to complete the survey to the best of their ability on the basis of the instructions provided.

After completing the PRO-CTCAE survey, a semiscripted cognitive debriefing interview was conducted. Scripts, including the content and sequence of the probing, were similar to those used previously in Spanish, German, and Danish PRO-CTCAE cognitive interviewing studies.^16-18^ First, a series of questions was asked about the patient’s sociodemographic characteristics. This was followed by probes to evaluate each of the distinct components of the PRO-CTCAE items (eg, recall period of the past 7 days; comprehension of the attributes of frequency, severity, and interference with usual activities; the concept of “at its worst”; and the various response options). The subset of PRO-CTCAE items that a respondent marked as posing difficulties for them during survey completion and the subset of PRO-CTCAE symptom terms prespecified within each of the eight scripts were both probed. Participants were queried about comprehension, clarity, relevance, inclusiveness, cultural appropriateness, and the cognitive processes used to generate responses. Probes elicited the respondent’s interpretations of the PRO-CTCAE symptom terms, terminology for the attributes (eg, frequency, severity), response choices, and phrasing of “at its worst” to allow subsequent evaluation of the equivalence between Korean and English PRO-CTCAE items. Interviewers probed any spontaneous patient comments about the comprehensibility or clarity of the questions or response choices; hesitations and/or body language or facial expressions that might indicate a problematic reaction to the items were also noted. Respondents were asked an open-ended question about whether they felt that there was anything else that should be added or changed in the questionnaire. The interviewer kept field notes documenting participant comments; interviews were audio recorded and transcribed for the analysis.

### Iterative Cycles of Analysis and Retesting

For analysis of the individual PRO-CTCAE items, interview field notes and transcripts were compiled, abstracted, and summarized item by item. Participants’ interview data were examined to gauge semantic and conceptual equivalence to the English PRO-CTCAE; participant responses to probing were categorized into linguistic themes (comprehension, relevance, inclusiveness, cultural appropriateness, and cognitive processes). Interview data pertaining to components of the PRO-CTCAE item stem (ie, symptom attributes, 7-day recall, and “at its worst” phrasing) and response options were analyzed and summarized across participants. The expert group reviewed the results of each round of data analysis. The proportion of respondents exhibiting any level of difficulty or hesitation with an item or with an item stem or response option was tabulated. PRO-CTCAE-Korean items that elicited difficulties in 10% or more of participants in round 1 interviews were flagged for expert group review and were considered for revision and retesting in rounds 2 and 3. Although a threshold of 20% or more of respondents with comprehension difficulties has been used in other PRO-CTCAE studies, we were also interested to explore PRO-CTCAE items for which comprehension difficulties were experienced by 10% to 20% of respondents because attention to the difficulties of respondents within this lower threshold might reveal opportunities to strengthen comprehension and ease of response. In addition, items recommended for additional testing by the expert group after reviewing round 1 data were also tested in both rounds 2 and 3. Item revision was considered by the study team on the basis of a detailed review of participant responses and in the context of their demographic characteristics, such as age, sex, or educational attainment, in an effort to produce a final Korean-language item library that would be well comprehended by diverse respondents, including those who are older and have lower educational attainment. Rounds 2 and 3 included testing of revised items using a methodology similar to that used in round 1.

## RESULTS

There were no statistically significant differences with respect to the sociodemographic and clinical characteristics of the participants included in each interview round (n = 40 per round; Table 1). Participants’ mean age was 55.4 years (standard deviation, 11.6 years), 86% were married, and approximately half (57%) had less than a high school education. Breast cancer (28%) and GI cancer (28%) were the most commonly reflected disease sites among study participants, followed by lung cancer (10.0%) and head and neck cancer (10.0%). The sample was diverse with respect to cancer stage (12% had stage I, 29% had stage II, and 32% had stage III disease); a little more than 70% of patients had an Eastern Cooperative Oncology Group performance status of 1 or 2. None of the participants reported their health as excellent; 35% and 34% of the participants reported their health as good and fair, respectively. The average time required to complete each PRO-CTCAE item was 10 seconds (standard deviation, 4.1 seconds; range, 4 to 25 seconds). With respect to the comprehension index, 72.5% of the participants in round 1 had a comprehension index greater than 90%, and in rounds 2 and 3, 95% or more of the respondents had a comprehension index greater than 90%.

**TABLE 1:**
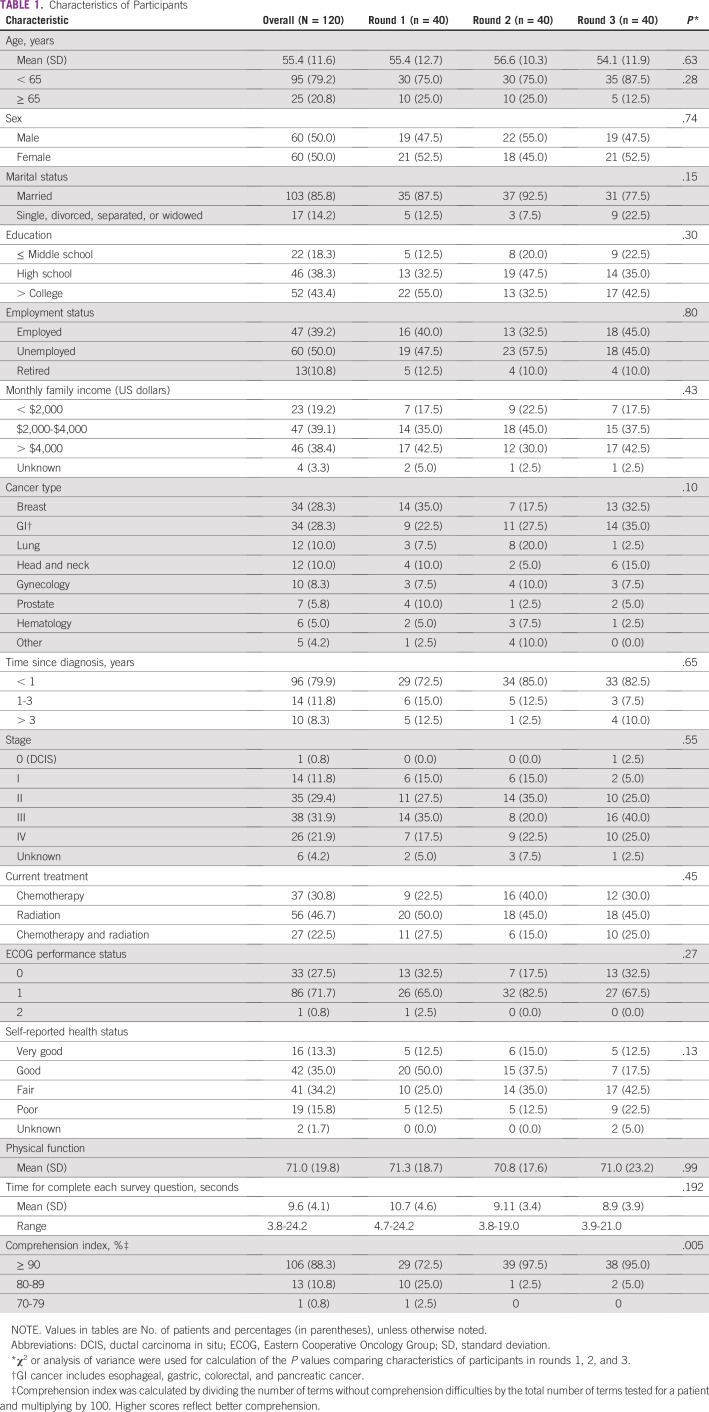
Characteristics of Participants

Overall, cognitive debriefing revealed that participants generally comprehended well the Korean-language phrasing of PRO-CTCAE symptom terms. In round 1, seven PRO-CTCAE-Korean symptom terms presented difficulties among 20% or more of participants. “Vaginal dryness” (difficulty in three [60%] of five participants), “nothing could cheer you up” (difficulty in 21 [52%] of 40 participants), and “pain, swelling, redness at a site of drug injection or IV [intravenous line]” (difficulty in three [33%] of nine participants) were the symptom terms that posed the greatest difficulties for respondents. This was followed by “wheezing” (difficulty in three [27%] of 11 participants), “pain” (difficulty in nine [22%] of 40 participants), “difficulty swallowing” (difficulty in two [22%] of nine participants), and “frequent urination” (difficulty in two [22%] of nine participants). An additional seven terms presented comprehension difficulties for 10% or more but less than 20% of participants (Table 2). Along with terms that presented difficulties in 10% or more of participants, the expert group recommended continued testing of two terms (“unusual vaginal discharge” and “loss of control of urine [leakage]”) in both round 2 and round 3. These two PRO-CTCAE symptom terms share terminology with two items that posed difficulties for 20% or more of respondents, specifically “vaginal dryness” and “frequent urination.” Thus, the expert group wished to confirm that there was acceptable comprehension of all PRO-CTCAE symptom terms using these terminologies (Table 2).

**TABLE 2:**
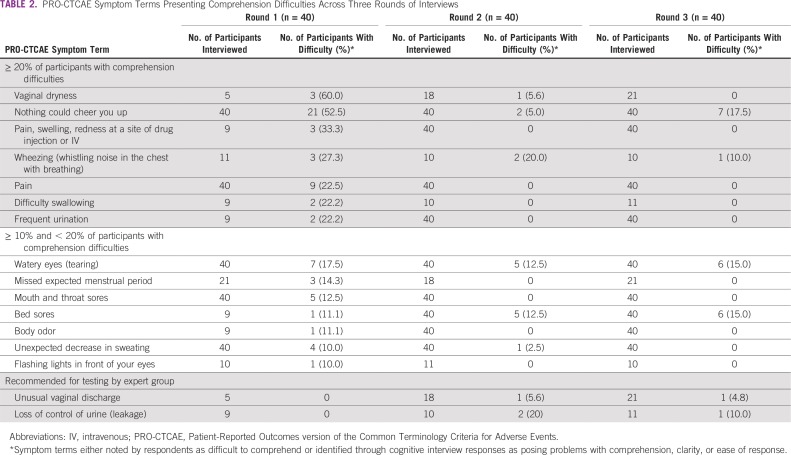
PRO-CTCAE Symptom Terms Presenting Comprehension Difficulties Across Three Rounds of Interviews

Cognitive interviewing highlighted some specific issues with the Korean-language translation. For example, participants experienced difficulties understanding specific terms such as “vagina,” “sore,” or “flashing.” In addition, participants had some difficulties with the clarity of phrasing of specific PRO-CTCAE items. For example, participants did not understand the meaning of “nothing” from the PRO-CTCAE-Korean symptom term “nothing would cheer you up” (Table 3), and participants were uncertain regarding whether “pain” was referring to overall body pain or to a site-specific pain such as pain in the hip or shoulder. In addition, some participants expressed difficulties with ease of judgement, recall, and response. Participants were confused about which injection site was meant by the term “pain, swelling, redness at a site of drug injection or IV,” and they were uncertain about whether the question was asking about pain, swelling, or redness at the site of venipuncture. A few participants experienced difficulty choosing a response for the PRO-CTCAE item asking about “missed expected menstrual period” when menstrual periods had recently become irregular as a result of treatment-induced menopause. In addition, the distinction between “not applicable” and “no” as response choices for this item was confusing for women who were already menopausal (Table 3).

**TABLE 3:**
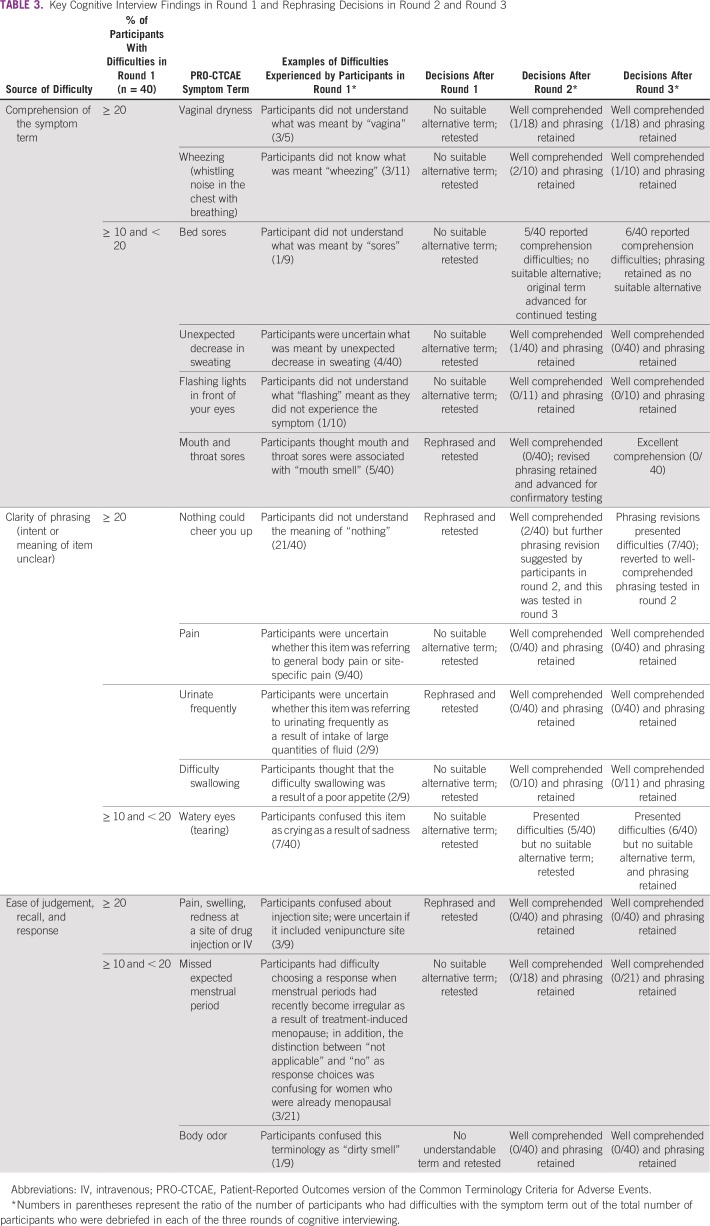
Key Cognitive Interview Findings in Round 1 and Rephrasing Decisions in Round 2 and Round 3

After round 1, phrasing for four PRO-CTCAE symptom terms (“nothing could cheer you up,” “frequent urination,” “pain, swelling, redness at a site of drug injection or IV,” and “mouth and throat sores”) was revised and tested with all participants in rounds 2 and 3. A total of 12 PRO-CTCAE-Korean terms were recommended for additional testing without any modification by the expert group, because no suitable phrasing alternatives could be identified by either the expert group or the patients interviewed in round 1. Given that four symptom terms (“vaginal dryness,” “bed sores,” “unusual vaginal discharge,” and “body odor”) were associated with some comprehension difficulties but had only been tested in a relatively small number of participants, these symptom terms were recommended for additional testing in rounds 2 and 3 to strengthen confidence in our conclusions that these items were adequately comprehended. No participants in round 1 experienced difficulties with the components of the PRO-CTCAE item stems, including phrasing related to symptom attributes (e.g., severity, frequency, interference), item response options, “at its worst” phrasing, and the 7-day recall period, and thus no changes to these elements were deemed necessary.

After round 2, only two terms (“wheezing” and “loss of control of urine”) presented continuing difficulties among 20% or more of participants, and in round 3, there were no PRO-CTCAE-Korean items that were reported as difficult to comprehend by 20% or more of participants (Table 2). However, after 17% of the participants (seven of 40 participants) had persistent difficulty understanding the revised Korean phrasing for “nothing could cheer you up” in round 3, the expert group decided to revert back to the phrasing that had been originally tested in round 2. The final version of the PRO-CTCAE-Korean was approved by the US NCI and is publicly available at their Web site (https://healthcaredelivery.cancer.gov/pro-ctcae/pro-ctcae_korean.pdf).

## DISCUSSION

In this study, we describe the translation and linguistic validation of the PRO-CTCAE item library in the Korean language. A majority of the items in PRO-CTCAE-Korean were well comprehended by Korean speakers in the first round of cognitive debriefing. After revisions and retesting, no item presented difficulties in 20% or more of participants tested in round 3. All participants expressed an accurate understanding of the concepts of frequency, severity, and interference. On the basis of our cognitive interview findings, we conclude that the PRO-CTCAE-Korean can be administered to Korean-speaking patients participating in cancer clinical trials, including those participants who are older or have lower levels of educational attainment.

Although we conducted an extensive translation process with multiple reconciliation meetings by the expert group, some PRO-CTCAE symptom terms were challenging to translate from English to Korean. First, certain words and phrases are not commonly used in Korean, such as “(un)expected” or “urge,” making accurate translation into Korean cumbersome. Second, some technical medical terms were difficult to translate into plain language, especially terms warranting Chinese characters that require a high level of health literacy. For example, some respondents did not know what was meant by “vagina” or “bed sores” in Korean; other investigators have made similar observations when testing PRO measures that have been translated into Korean.^19^

Respondents indicated that some PRO-CTCAE questions were difficult to interpret or had a somewhat unclear meaning or intent. For example, respondents were uncertain about the context surrounding their response to specific items, asking, for example, whether the PRO-CTCAE pain items should be interpreted as asking about generalized body pain or a site-specific pain. Respondents were also uncertain whether the questions about “watery eyes” referred to crying as a result of feeling sad. Such lack of clarity might have occurred because the phrasing of these PRO-CTCAE questions does not address such specific contextual considerations and thus leaves room for respondents to interpret the meaning of the questions somewhat differently. Fortunately, such instances were rare in this large and diverse sample.

During cognitive interviews, the PRO-CTCAE-Korean symptom term with the most comprehension difficulties was “nothing could cheer you up”; this phrasing had two challenges. First, participants did not understand the meaning of “nothing” in Korean. Second, the translated phrasing included a double-negative expression. Because there are no double-negative expressions in Korean, the term sounded awkward when translated into Korean. Notably, a similar issue has been reported in another linguistic validation study.^19^ To improve comprehension, a modification was made from “nothing could cheer you up” to “anything could not cheer you up.” However, patients also found this phrasing cumbersome because the word “anything” is not a common expression in Korean. After discussion with the expert group and the US NCI, we decided to include in parentheses an elaboration with some examples of the kinds of things (e.g., a visit from family) that might ordinarily serve to elevate one’s mood.

As noted with both the German- and Spanish-language linguistic validation studies,^17,18^ we also observed that participants had more difficulty comprehending symptomatic AEs they had not experienced. For example, respondents who had experienced certain less common symptomatic AEs such as body odor were able to accurately report them using the proposed phrasing, whereas other respondents who were somewhat uncertain about the intent or meaning reported that they had difficulty responding because they had not had such experiences.

Both the strengths of this study design as well as several caveats should be considered when interpreting our findings. First, because our study was conducted with a sample of patients currently receiving cancer treatment and because the interviews lasted 30 to 60 minutes, participants had to have sufficient physical and emotional stamina to complete study-related procedures. Thus, our sample may have been biased toward inclusion of participants with preserved performance status. However, of note, 15% of our sample reported their health status as poor, and an additional 35% reported their health status as fair. Second, this study was conducted at a single cancer center, and all study participants were residents of South Korea. Although we believe the size and the clinical and demographic diversity of our sample provide rigorous evidence of the content validity of PRO-CTCAE-Korean, additional testing to confirm its comprehensibility and cultural acceptability to Korean speakers who reside in other countries could be considered. At the same time, there is some evidence to support the generalizability of findings from PRO linguistic validation studies to Korean speakers residing both in Korea and in the United States.^20^ Third, although our sample was quite diverse with respect to tumor type, some specific tumor subgroups, such as those with brain or CNS tumors, were under-represented in the sample. This limitation might be important because patients with those tumors may suffer from cognitive deficits that could affect comprehension of self-report measures. Finally, in this study, PRO-CTCAE items were tested using paper questionnaires, whereas administration of PRO-CTCAE may be electronic in some trials. Measurement equivalence between paper, Web, and automated telephone administration of PRO-CTCAE-English items has been demonstrated^21^ and could also be explored in future studies that use PRO-CTCAE-Korean.

However, confidence in the generalizability of these findings is strengthened by the rigorous process of translation and cognitive interviewing and the inclusion of a large, demographically and clinically diverse sample of patients with cancer. A quantitative validation study to evaluate the psychometric properties and responsiveness of PRO-CTCAE-Korean in a large sample of Korean speakers undergoing cancer treatment is in progress.

The overall goal of the Korean-language version of the PRO-CTCAE is to facilitate an improved understanding of the patient experience of symptomatic toxicity to better inform decisions by patients, clinicians, and policymakers in Korea. As the number of cancer clinical trials in Korea increases,^22,23^ the availability of PRO-CTCAE-Korean allows PRO data about symptomatic toxicities to be gathered in trials and thus to reflect the experiences of a diverse population.

## Figures and Tables

**TABLE A1:**
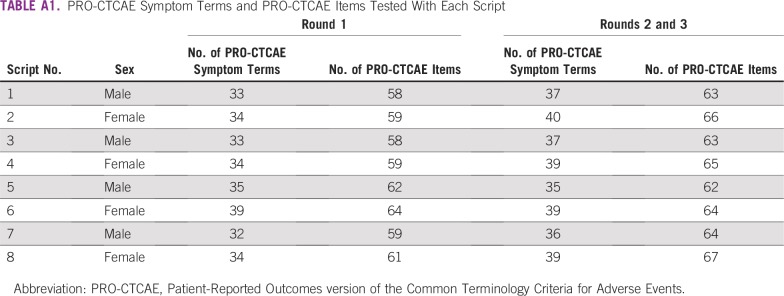
PRO-CTCAE Symptom Terms and PRO-CTCAE Items Tested With Each Script

## Data Availability

The following represents disclosure information provided by authors of this manuscript. All relationships are considered compensated. Relationships are self-held unless noted. I = Immediate Family Member, Inst = My Institution. Relationships may not relate to the subject matter of this manuscript. For more information about ASCO's conflict of interest policy, please refer to www.asco.org/rwc or ascopubs.org/jco/site/ifc. **Honoraria:** Menarini, Amgen, Pfizer, AstraZeneca, Roche, Janssen, Merck Sharp & Dohme, Bristol-Myers Squibb-Ono Pharmaceutical, Eisai **Consulting or Advisory Role:** Boehringer Ingelheim, Samsung Bioepis **Honoraria:** Pfizer No other potential conflicts of interest were reported.
